# Evaluating clinical communication skills of medical students, assistants, and professors

**DOI:** 10.1186/s12909-023-05015-4

**Published:** 2024-01-03

**Authors:** Masoumeh Moezzi, Sara Rasekh, Elahe Zare, Masoud Karimi

**Affiliations:** 1 Department of Community Medicine, School of Medicine, Social Health Determinate, Shahrekord University of Medical Sciences, Shahrekord, Iran; 2 Shahrekord University of Medical Sciences, Shahrekord, Iran; 3 Department of Health, Promotion, School of Health, Student Research Committee, Shiraz University of Medical Sciences, Shiraz, Iran; 4 Research Center for Health Sciences, Institute of Health, Department of Health Promotion, School of Health, Shiraz University of Medical Sciences, Shiraz, POBox: 71645-111, Iran

**Keywords:** Clinical communication skills, Knowledge, Attitude, Performance, Medical students, Assistants.

## Abstract

**Background:**

The skill of communicating with the patient is one of the basic clinical skills and part of the necessary competencies for medical doctors. The present study aimed to evaluate the knowledge, attitude, and performance (KAP) of medical students, assistants, and professors of Shahrekord University of Medical Sciences regarding clinical communication skills with patients.

**Method:**

This cross-sectional study was conducted at the hospitals of Shahrekord University of Medical Sciences in Iran. The study included a total of 289 participants, consisting of 51 professors, 72 assistants, 90 external staff, and 76 interns who work at these hospitals. The participants were selected through a convenience sampling method. The data-gathering tools used included a questionnaire to collect demographic characteristics, a researcher-made questionnaire to assess knowledge, a communication skills attitude questionnaire, and a communication skills survey questionnaire. The data were analyzed using descriptive statistics such as mean, standard deviation, and frequency, as well as statistical tests that included one-way ANOVA and Pearson’s correlation test. The significance level for the study was considered to be 0.05.

**Results:**

The mean scores of knowledge of professors were higher compared to other groups (*P* = 0.002). All participating groups had a positive attitude toward learning communication skills. There were statistically significant differences between the mean scores of the communication performance of the study groups (*P* < 0.001). There was a positive relationship between positive attitude and communication performance, and a significant negative relationship was observed between negative attitude and communication performance.

**Conclusion:**

The results indicate the relatively favorable attitude and performance of the groups and their low knowledge. It is suggested that the doctor-patient communication skills courses be included as one of the necessary courses in the medical education curriculum.

## Introduction

Effective communication is an essential and multidimensional aspect of purposeful exchange, which cannot be avoided [1]. Communication skills are considered crucial competencies for medical doctors, as per expert opinions. Despite their complexity, these skills can be learned and taught [2, 3]. One of the fundamental principles of professionalism in medicine is the commitment to proper communication with patients, based on an understanding of moral and legal issues [4].

Effective communication between doctors and patients is crucial for quality medical care. Poor communication can negatively impact various aspects of medical care, including history taking, disease diagnosis, and the use of effective treatments. Studies have shown that patients’ stresses and worries can have adverse effects on their health. Therefore, physicians should establish proper communication and consider patients’ feelings and concerns to provide more useful and effective medical care [5]. Evidence indicates that physicians often lack adequate knowledge of communication principles, resulting in significant issues in doctor-patient interactions [6]. In many cases, doctors’ poor communication causes patient complaints, not their scientific abilities [7, 8]. According to Shiraly et al.‘s study, despite having good knowledge about communication skills with their patients, most doctors reported inadequate performance. It seems that doctors’ knowledge is not effectively applied, leaving the problem unresolved [9]. The researchers evaluated the communication skills of 53 internal assistants in a clinical examination station and found that most of them displayed poor communication skills [10]. Therefore, it is necessary to provide medical doctors with proper and systematic training in patient communication skills [7]. However, Understanding medical students’ attitudes towards communication skills training is important as it can influence their communication behaviors in clinical settings [11]. By gaining insights into their beliefs and attitudes towards doctor-patient communication, we can improve the effectiveness of communication skills training programs [12]. Therefore, communication skills teaching has become a vital topic in medical education curriculums across many countries, where medical students are required to pass communication skills training courses before starting clinical education. These courses typically cover methods of taking history and conducting medical interviews [13]. However, despite the growing importance of communication skills in medical education, these skills are not included in the official program of medical education in Iran. Instead, students’ learning is mostly based on indirect and experimental modeling from their senior students and professors, with only scattered movements made at the level of some universities of medical sciences [14, 15]. As a result of in-depth interviews with medical students in Iran, it was found that they did not believe in their ability to create effective relationships with patients, indicating that educational programs have not been efficient in creating effective relationships with patients. Therefore, there is still a need to teach communication skills and evaluate the effectiveness of training programs [10]. It was also reported that patient satisfaction with the communication skills of patients and medical assistants was average. Therefore, it is recommended that the training and evaluation of communication skills be a central part of the assistants’ formal curriculum [16].

Most of the previous studies on the communication skills of clinical care providers have focused on nurses and medical students [17–21]. Fewer studies have examined the communication skills of university professors and their assistants [9, 22], and none have compared the communication skills of medical students, assistants, and professors. Additionally, most studies have mainly evaluated participants’ performance, with less attention paid to their knowledge and attitude [23]. Research reveals that interventions addressing the cognitive, emotional, and psychomotor domains of behaviors are more effective in improving communication skills. Knowledge, Attitude, and Performance (KAP) studies are crucial in designing such interventions [24]. Therefore, this study aims to assess the knowledge, attitudes, and performance of medical students, assistants, and professors in terms of clinical communication skills with patients.

## Methods

This cross-sectional study was conducted on medical students (externs and interns), assistants, and professors of the teaching hospitals of Shahrekord University of Medical Sciences, Iran in 2019. The participants in this study were chosen using the convenience sampling method. To achieve this, all 120 clinical professors and their 87 assistants were considered, and finally, 51 professors and 72 assistants completed the questionnaires. The sample size of 185 medical students was determined based on Krejcie & Morgan’s (1970) table for determining the sample size from a given population (which was 340 for this study) [25]. To accomplish this, 100 externs and 85 interns were randomly selected from a total of 180 externs and 160 interns. The inclusion criteria for this study were medical students who had completed the entire training course at Shahrekord University of Medical Sciences and were not transfers, medical assistants in clinical fields studying at Shahrekord University of Medical Sciences who were not guests or transferees, all clinical professors of Shahrekord University of Medical Sciences, and participants who had provided their consent to participate in the study. Exclusion criteria included dissatisfaction with participating in the study and failure to respond to the questionnaire.

Data were collected through a demographic information form (including sex, age, marital status, and education level), a questionnaire for measuring knowledge about communication skills, a questionnaire for measuring attitude towards communication skills, and a questionnaire for measuring communication performance.

Knowledge was assessed by a researcher-made questionnaire consisted of 10 multiple-choice questions, the correct answer to each question was given a score of one, and the incorrect answer (or I don’t know) to each question was given a score of zero, so, the possible score range was between zero and 10. The knowledge score was categorized into two levels based on the 50% of highest possible score, indicated by low (0–5), and high [6–10]. The face and content validity of the questionnaire was confirmed by a panel of 10 PhDs in health promotion and medical education. To evaluate the content validity of the questionnaire, opinions of a panel of 10 health education and promotion professionals were considered, and subsequently, the content validity ratio (CVR) and the content validity index (CVI) were calculated. The CVR for all items in the questionnaire was higher than 0.78, which met the acceptable criteria based on Lawshe’s criteria [26]. Additionally, the CVI for all items was over 0.79, which was also acceptable based on the criteria set by Waltz and Bussel [27]. Test-retest method on a pilot sample of 30 subjects with two weeks’ intervals was used for assessing external reliability of the questionnaire, and the correlation coefficient of 0.75 (*P* = 0.003) confirmed its reliability.

The participants’ attitudes toward doctor-patient communication skills was assessed using a standard Communication Skills Attitude Questionnaire (CSAS) which was designed and validated by Rees et al. (2002) in England [28]. This questionnaire has 26 items with a five-point Likert scale (completely agree-completely disagree) and includes two parts: a positive attitude (13 items) and a negative attitude (13 items) towards doctor-patient communication skills. A higher score in each section indicates a more positive and negative attitude toward the importance of doctor-patient communication skills. The Persian version of this questionnaire was validated in 2017 by Yakhforoshha et al. [29]. In the current study, the internal reliability of the questionnaire was measured by calculating the Cronbach’s alpha coefficient, and Cronbach’s alpha coefficients of 0.90 and 0.70 for the positive and negative attitude questionnaires, respectively, indicating the excellent and acceptable reliability of this questionnaire. In examining the external reliability of the questionnaire with the test-retest method on a pilot sample of 30 subjects with two weeks’ intervals, the correlation coefficient of 0.85 (*P* = 0.001) indicates the appropriate reliability of the questionnaire.

The participant’s performance was measured using communication skills survey questionnaire which was designed and validated by Javaher et al. (2014) in the Persian language [30]. This questionnaire includes 28 items with a five-point Likert scale (always-never) with a possible score range of 28 to 140. The higher score indicated a better performance in the relationship with the patient. The performance score was categorized into two levels based on the 50% of highest possible score, indicated by low (< 70), and high (≥ 70). Cronbach’s alpha coefficient of 0.75 indicated the acceptable internal reliability of the questionnaire. In examining the external reliability of the questionnaire with the test-retest method on a pilot sample of 30 subjects with two weeks’ intervals, the correlation coefficient of 0.81 (*P* = 0.001) indicated the appropriate reliability of the questionnaire.

After explaining the aims of the study to four groups of participants and signing the informed consent form by them, they were included in the study and questionnaires were administered to them. It took, on average,30 min to complete the questionnaires and the researcher tried to administer the questionnaires in the times when the work load of participants was low, so they could respond easily and without worrying about work. It should be noted that due to the busy schedule of the professors, the questionnaire was provided to all professors in some other online ways such as email and social networks, too.

The data were analyzed using SPSS24 software. Demographic information was reported using frequency distribution, mean and standard deviation. The Kolmogorov-Smirnov test with the Bonferroni post hoc test was used to examine the normality of data distribution. Pearson’s correlation coefficient was used to compare mean scores of knowledge, attitude, and performance. One-way ANOVA was used to compare means on more than two groups. The significance level was set at < 0.05.

## Results

Based on the sample size which was considered for this study, 392 questionnaires were administered to the participants and 289 people completed the questionnaires (total response rate = 73.7%). The lowest response rate was related to professors (42.5%) and assistants (82.7%), interns (89.4%) and interns (90.0%) were in the next ranks. Among the study participants, 125 (43.3%) were male and 164 (56.7%) were female. Most of the participants were single (51.6%) and about 39.7% of them were married, while 8.7% of the participants did not answer the marital status question. Table 1 shows the mean age of study participants in four education level groups by sex. (Table 1)

**Table 1:** Mean age of participants by sex and education level

Education level	N	Male		Female	
		Mean(years)	SD	Mean(years)	SD
Professor	51	52.71	5.05	46.38	6.12
Assistant	72	36.11	4.40	33.80	3.66
Intern	76	25.70	3.30	25.50	1.67
Extern	90	25.52	3.36	24.40	1.42

As shown in Table 2,, the knowledge mean scores of all groups participating in the study were in the low category. The highest knowledge score was related to professors, significantly different from other studied groups (*P* = 0.002). There were no significant differences in the positive and negative attitudes of the participants in the study towards communication skills among the different study groups. The findings of the study showed that, in general, the mean scores of communication performance in all the participating groups were in the high category, and the communication performance of the professors was significantly better than the other studied groups (*P* < 0.001), besides, the performance of externs was significantly better than assistants (Table 2).

**Table 2:** Comparing the mean score of knowledge, attitude, and performance of participants between for education groups

Variable	Education group	Range of scores	Mean	SD	F	P*
knowledge	Professor	0–6	4.04	1.28	5.064	0.002
	Assistant	0–7	3.44	1.31		
	Intern	1–6	3.23	1.32		
	Extern	0–6	3.21	1.24		
Positive Attitude	Professor	50–62	57.61	2.95	1.257	0.289
	Assistant	47–65	59.01	3.99		
	Intern	43–65	58.57	4.06		
	Extern	43–65	58.63	4.68		
Negative attitude	Professor	24–41	31.70	3.81	0.312	0.816
	Assistant	24–44	32.43	4.24		
	Intern	19–47	31.94	4.58		
	Extern	21–52	32.23	5.39		
Performance	Professor	106–137	115.35	5.17	10.016	< 0.001
	Assistant	88–130	107.95	7.58		
	Intern	86–130	109.94	8.88		
	Extern	95–132	111.04	7.85		

*One-way ANOVA

In examining the correlation between the studied variables, it was found that there were no significant correlations between the knowledge mean scores with positive attitude, negative attitude, and communication performance in any of the studied groups. In the group of professors, no significant correlation was observed between any of the studied variables, but in the other three groups significant positive relationships were observed between positive attitude and communication performance, that is, the more positive the attitude toward doctor-patient communication skills, the better the performance. Negative significant correlations were observed between positive attitude and negative attitude in all three groups, that is, the higher the positive attitude, the lower the negative attitude towards the importance of patient communication skills. In the groups of assistants and externs, significant negative correlations were seen between negative attitude and communication performance, which means that the more negative attitude about the importance of communication skills, the weaker communication performance was reported. In almost all cases, the correlation coefficients were less than 0.05, which means that the observed correlations were weak (Table 3).

**Table 3:** Pearson correlation coefficients between the mean score of knowledge, attitude, and performance of participants in four education groups

Education Group	Variable	knowledge	Positive attitude	Negative attitude
Professor	Knowledge	1		
	Positive attitude	-0.213	1	
	Negative attitude	-0.132	0.268	1
	Performance	0.098	0.029	-0.144
Assistant	Knowledge	1		
	Positive attitude	0.146	1	
	Negative attitude	0.021	-0.426**	1
	Performance	-0.038	0.229*	-0.246*
Intern	Knowledge	1		
	Positive attitude	0.108	1	
	Negative attitude	0.041	-0.429**	1
	Performance	-0.105	0.349**	-0.177
Extern	Knowledge	1		
	Positive attitude	-0.081	1	
	Negative attitude	0.175	-0.531**	1
	Performance	-0.093	0.321**	-0.258

*p < 0.05; **p < 0.001

## Discussion

The results of this study showed that the knowledge status of all participated groups (externs, interns, assistants, and professors) were in the low level (based on the 50% of possible achievable score), and the knowledge score of professors was significantly higher compared to assistants, interns, and externs, but the knowledge score of assistants, interns, and externs were not significantly different. Since communication skills are not officially taught in the educational curriculum of medical students in Iran, this difference may be due to more experiences of professors than assistants and students. These results were consistent with the results conducted by Wang et al. (2022) and Güner et al. (2019) [31, 32], it seems that since, based on the available evidence, communication skills are mainly acquired and can be learned [33], medical students, assistants, and professors need to participate in doctor-patient communication skills courses.

In our study, medical students, assistants, and professors have a positive attitude toward learning communication skills, while, there were no significant differences between the different study groups, however, the positive attitude of the assistants, interns, and externs was slightly more than that of the professors. Since formal communication skills training is not given to medical students in Iran, this difference may be due to the fact that students and assistants spend more time with patients than professors and feel a greater need to learn communication skills. The results of Timilsina et al. (2019) and Ratnaprabha et al. (2021) were in line with the findings of the present study [34, 35]. Although, in the groups of assistants, interns, and externs, a significant inverse relationship was observed between negative attitude and communication performance. However, the results of a longitudinal study showed that there is a possibility of increasing negative attitudes in the long term, and it is suggested that in order to prevent attitudes from worsening over time, communication skills training should be continuous or at least at intervals of less than 6 months [36].

The results of the current study indicate that the communication performance of all four groups were in the high level (based on the 50% of possible achievable score). There were significant differences between the communication performance scores of the different groups, with the professors having a higher mean score compared to the assistants, interns, and externs. Which may be because of their more experiences. However, no similar study comparing the communication performance of different groups was found. Different studies that utilized the same tool to measure doctors’ communication skills produced varying results. For example, a cross-sectional study by Rezaiyan et al. (2015) found the mean score of the communication skills of the academic staff at Rafsanjan University of Medical Sciences to be 92.56 ± 7.25 (out of the range of 34–179), which was at an average level [22]. In another study by Peyman et al., the overall average of the communication skills of the faculty members at Ilam University of Medical Sciences was 106.53 ± 8.59. In a similar study by Attarha et al. in Arak University of Medical Sciences, this value was reported as 121.8 ± 8.8 [37]. The discrepancies in the results of these studies may be attributed to differences in the study populations in terms of various variables such as age, sex, work experience, education level, and receiving training regarding clinical communication skills.

Research suggests that trained physicians possess better targeted communication skills [38], which in turn leads to more accurate diagnosis and treatment, as well as higher patient satisfaction rates. Therefore, it is imperative to educate both students and professors to improve communication performance. However, studies conducted by Beaird et al. (2017) and Dong et al. (2015) show that there is no significant correlation between knowledge scores and attitude, negative or positive, or communication performance in any of the studied groups [39, 40]. This indicates a need for educational programs that go beyond merely enhancing knowledge and are presented in innovative and interactive ways.

In this study, we compared four groups (externs, interns, assistants, and professors), which is a unique approach. However, we faced some limitations, including a low response rate from the professors despite multiple methods being used to encourage them to complete the questionnaire. Another limitation is that the questionnaire was self-administered, and we couldn’t observe the participants’ communication skills when dealing with patients. Therefore, the responses may be biased by social desirability.

## Conclusion

According to the results of the present study, professors, medical students and assistants had average knowledge about clinical communication skills, but their performance and attitude were above average and relatively favorable. Also, the skills and knowledge of professors were more compared to assistants and general medical students, which shows that one of the ways to improve communication skills is to create and feel the need to establish proper communication with patients after increasing experience and length of service. However, the lack of knowledge of professors and assistants regarding communication skills, despite their professional experience in the community, indicates that being in the community and professional activity alone is not enough to acquire communication skills, and the need for training is felt.

## Data Availability

The data that support the findings of this study are available on request from the corresponding author. The data are not publicly available due to their containing information that could compromise the privacy of research participants.
